# Fine Definition of the CXCR4-Binding Region on the V3 Loop of Feline Immunodeficiency Virus Surface Glycoprotein

**DOI:** 10.1371/journal.pone.0010689

**Published:** 2010-05-18

**Authors:** Qiong-Ying Hu, Elizabeth Fink, Yang Hong, Cathy Wang, Chris K. Grant, John H. Elder

**Affiliations:** 1 Department of Immunology and Microbial Science, The Scripps Research Institute, La Jolla, California, United States of America; 2 Custom Monoclonals International, West Sacramento, California, United States of America; Institut Pasteur, France

## Abstract

The chemokine receptor CXCR4 is shared by primary and laboratory-adapted strains of feline immunodeficiency virus (FIV) for viral entry. Our previous studies implicated a contiguous nine-amino-acid region of the V3 loop of the FIV envelope surface as important in CXCR4 binding and virus entry. The binding is specific for CXCR4 since it can be inhibited by AMD3100, a selective CXCR4 inhibitor. Additional site-directed mutagenesis was used to further reveal the key residues. Binding studies indicated that basic residues R395, K397, R399 as well as N398 are critical for CXCR4 binding. The effect of other amino acid residues on receptor binding depends on the type of amino acid residue substituted. The binding study results were confirmed on human CXCR4-expressing SupT1 cells and correlated with entry efficiency using a virus entry assay. Amino acid residues critical for CXCR4 are not critical for interactions with the primary binding receptor CD134, which has an equivalent role as CD4 for HIV-1 binding. The ELISA results show that W394 and W400 are crucial for the recognition by neutralizing anti-V3 antibodies. Since certain strains of HIV-1 also use CXCR4 as the entry receptor, the findings make the feline model attractive for development of broad-based entry antagonists and for study of the molecular mechanism of receptor/virus interactions.

## Introduction

Feline immunodeficiency virus (FIV) is the only nonprimate lentivirus that causes an AIDS-like disease in its natural host, the domestic cat [1]. Thus, FIV infection in cats has been established as a valuable animal model for the development of anti-retroviral agents against lentivirus including HIV, and study of lentiviral pathogenesis [2]–[5]. In regard to receptor usage, the two lentiviruses have a common mechanism, but they act through distinct binding receptors. HIV-1 uses CD4 as a primary binding receptor, whereas FIV utilizes CD134 [6], [7]. After interaction with the primary binding receptor [8], [9], however, FIV (primary and laboratory-adapted FIV strains [10]) and T-cell tropic HIV-1 strains both utilize the chemokine receptor CXCR4 as the entry receptor. The predicted amino acid sequence of feline CXCR4 displays 94.9% identity to human CXCR4, with the majority of the differences located in the N-terminus and the second extracellular loop [8]. In addition, it has been reported that the second extracellular loop of CXCR4 contains a critical determinant for the function of CXCR4 as a receptor for infection with FIV [11], [12]. Both human and feline CXCR4 have a number of negative charges at the extracellular surface [8], [13]–[16].

In contrast to the negative charged extracellular surface of CXCR4, the hypervariable region 3 (V3 loop) of HIV-1 is positively charged and binds to the surface of the receptor in the N-terminal extracellular loop [17]. HIV-1 V3 typically consists of 35 amino acids (range 31 to 39) and is functionally important [18]. The HIV-1 V3 loop has been previously termed as the “principal neutralizing determinant” of HIV-1, since many HIV-1 neutralizing antibodies from infected individuals target this region of gp120 [19]. Such antibodies prevent the binding of gp120 to the chemokine receptors and thus block the events leading to viral fusion [20], [21]. The findings indicate that the V3 amino acid sequence determines whether the virus binds to CCR5 (“R5 phenotype”) as a predominantly macrophage-tropic isolate, or to CXCR4 (“X4 phenotype”), which are primarily T cell-tropic isolates [20]–[22]. Moreover, the presence of a basic residue at V3 positions 306 or 322 is associated with X4 and dual-tropic phenotype (X4R5 viruses), whereas the presence of a neutral residue and a negatively charged residue at positions 306 and 322, respectively, is correlated with R5 viruses (the “11/25 rule”) [23]. Then, a new “11/24/25 rule” updates that: positively charged amino acids at positions 11, 24, or 25 define X4; otherwise the virus has a R5 phenotype [24]. Thus, the V3 loop is a primary target for HIV-1 entry inhibitors that are being developed as antiviral drugs [18].

Although the envelope glycoproteins of FIV and T-cell tropic HIV-1 share only minor sequence identity in SU, there are analogies in the location and distribution of the SU variable regions V3-V5 [25]–[30]. Although the consensus sequences of conserved cysteine residues between both viruses display a low degree of homology, there still exist some similarities. First, the FIV V3 loop has an approximate length of 41 amino acid residues (equivalent to HIV V3). Secondly, both FIV and HIV V3 regions are positively charged [9], [18], [24], [31], [32]. A JPRED analysis predicts the secondary structure of the V3 loops of both viruses as to display a high degree of similarity. Furthermore, both V3 loops are predicted to have a relatively conserved centrally located tip flanked by two beta sheets [33], [34]. Finally, the consensus sequence of the HIV-1 V3 tip is a relatively conserved GPGR or GPGQ [18]. Similarly, the amino acids at the tip of FIV V3 (namely the “N44 region” we describe in the present study) are conserved across strains, including two tryptophans (98 and 100% conserved, respectively), one lysine (55% conserved), and two arginines that are 73% and 99% conserved, respectively. The domain and amino acid residues of HIV-1 involved in CXCR4 binding have been clearly identified [17], [18], [24], [31], [32], [35], however, amino acid residues critical for SU/CXCR4 interacts with FIV still require mapping.

Our previous binding studies using SU-Fc deletion mutants and SU- specific monoclonal antibodies strongly support the involvement of the V3 loop of SU in the FIV-CXCR4 interaction. Furthermore, a contiguous nine-amino-acid region (SSWRQKNRW, designated N44) of FIV V3 is a neutralization target for CD134-dependent virus neutralization via blocking CXCR4 binding and virus entry [36].

The purpose of this study was to define the key amino acid residues required for CXCR4 binding and establish the relationship between binding and entry. We created a series of SU mutants by site-directed mutagenesis to introduce amino acid substitutions in the N44 region, together with a panel of V3-specific antibodies and then used these mutants to define residues critical for CXCR4 interaction.

## Results

### Detection of CD134 or CXCR4 expression in cell lines

**Figure 1:**
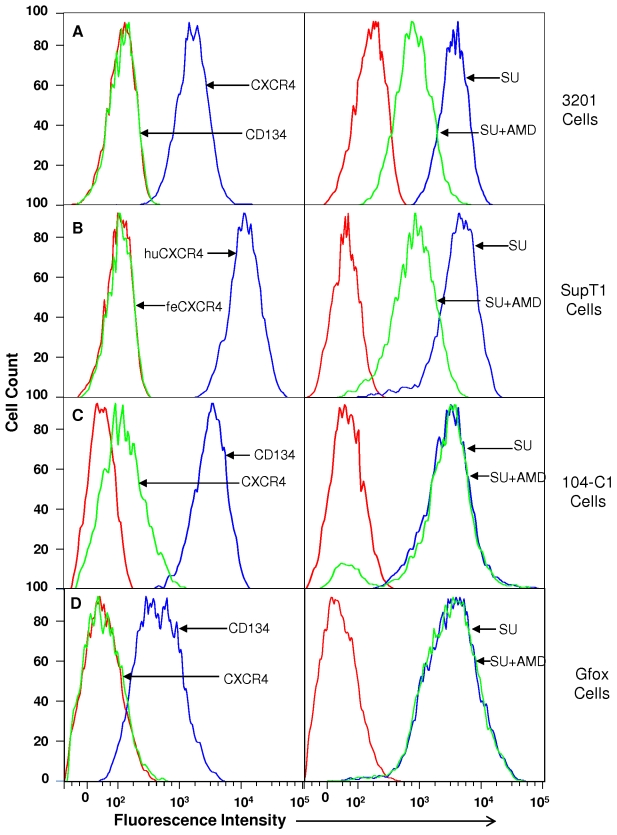
Identification of receptor expression on the cell surface and SU binding to cells of 3201, SupT1, 104-C1 and Gfox, respectively, detected by FACS analysis. The detection of CXCR4 and CD134 was carried out by using CXCR4 or CD134-specific antibody, followed by anti-mouse (CXCR4) or anti-rabbit (CD134) IgG conjugated with phycoerythrin. Binding of FIV SU-Fc to cells was measured by using a phycoerythrin-conjugated anti-Fc antibody, in the presence and absence of the CXCR4 antagonist AMD3100. Cells were incubated with SU-Fc or pre-treated with AMD3100 for 30 min prior to the addition of SU-Fc. Results are representative of three independent determinations.

To characterize the receptor binding properties of wild type and mutated FIV PPR SUs, we used a series of continuous and engineered cell lines (Figure 1). Fluorescence activated cell sorting (FACS) analysis of the cell surface expression of CD134 and CXCR4 showed that 3201 cells expressed relatively high levels of CXCR4 but were negative for CD134 (Figure 1A, left panel). FIV PPR SU-Fc bound strongly to 3201, but the binding was inhibited when pretreated with CXCR4 inhibitor AMD3100 (Figure 1A, right panel). For SupT1 cells which express human CXCR4 [37], we utilized a human specific anti-CXCR4 antibody, 12G5, which does not recognize feline CXCR4 [38]–[40]. SupT1 cells also expressed CXCR4 at a high level (Figure 1B, left panel) and PPR SU bound strongly to the human homologue, and the binding was competed by AMD3100 (Figure 1B, right panel). Remarkably, human CXCR4 is a more efficient receptor for FIV than either feline CXCR4 or rat CXCR4 [11]. By contrast, 104-C1 cells expressed a high level of CD134 but a low concentration of CXCR4 (Figure 1C, left panel). FIV-PPR SU bound well to this cell line via CD134 with no evident blocking by AMD3100 (Figure 1C, right panel), which is consistent with previous reports [6], [9], [41]. Gfox cells are CrFK cells that have been engineered to over-express CD134 [6]. Our results showed that feline CD134 was observed (Figure 1D, left panel) and SU-Fc specifically interacted with feline CD134 (Figure 1D, right panel). CXCR4 appeared insignificant on these cells (Figure 1D, left panel). However, Gfox cells are infectable with FIV field strains and infection is blocked by AMD3100, consistent with entry by CXCR4 expression [6]. Also, CrFK cells express CXCR4 RNA [8] and our quantitative real time PCR results indicated that Gfox cells express CXCR4 mRNA at levels comparable to CrFK cells (data not shown).

### Characterization of feline anti-CXCR4 antibody

**Figure 2:**
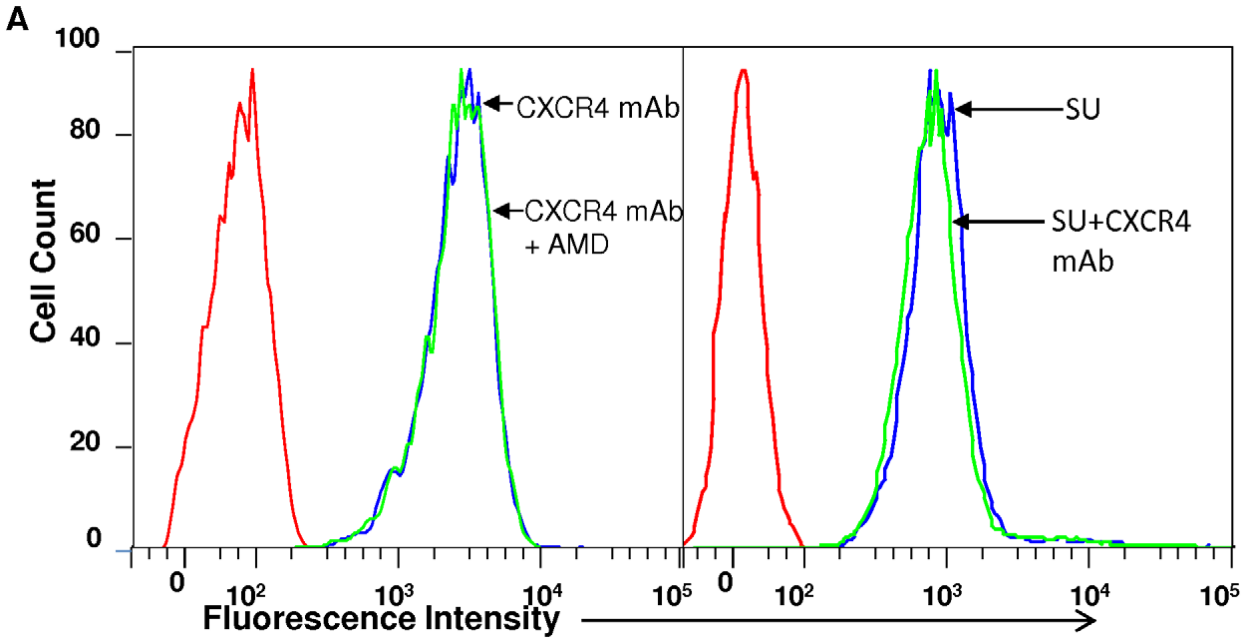
Analysis of inhibition of feline CXCR4 antibody binding to 3201 cells by pretreatment with AMD3100(left panel) or blockade of SU binding to 3201 cells by pretreatment with fCXCR4 mAb (right panel). Results are representative of three independent determinations.

The feline anti-CXCR4 antibody used in the present study had no cross-reactivity with human CXCR4 (Figure 1B, left panel). To determine whether the binding region of anti-CXCR4 antibody corresponded to CXCR4-binding domain for SU, we performed competition binding assay in 3201 cells. FACS analysis showed that AMD3100 did not block anti-CXCR4 antibody binding to 3201 cells and the antibody did not inhibit SU-Fc binding to 3201 cells (Figure 2), indicating the recognition epitope for the antibody was distinct from the binding site for SU.

### V3 is the major binding domain of SU for CXCR4 in 3201 cells

**Figure 3:**
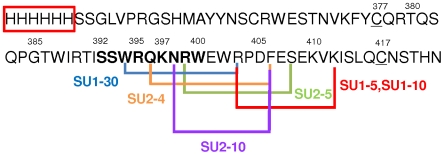
Sequence of His-tagged PPR V3 peptide and binding sites of anti-V3 antibodies on the V3 loop of FIV SU. Red box indicates 6XHis. The amino acids are numbered according to the sequence of the V3 loop of PPR SU, and the V3 loop is bordered by cysteine (C), which is underlined. N44 region of V3 loop is indicated in bold. Linear binding sites of V3 antibodies SU1-5, SU1-10, SU1-30, SU2-4, SU2-5 and SU2-10 are indicated respectively. SU1-7 is not shown due to its conformation dependence.

**Figure 4:**
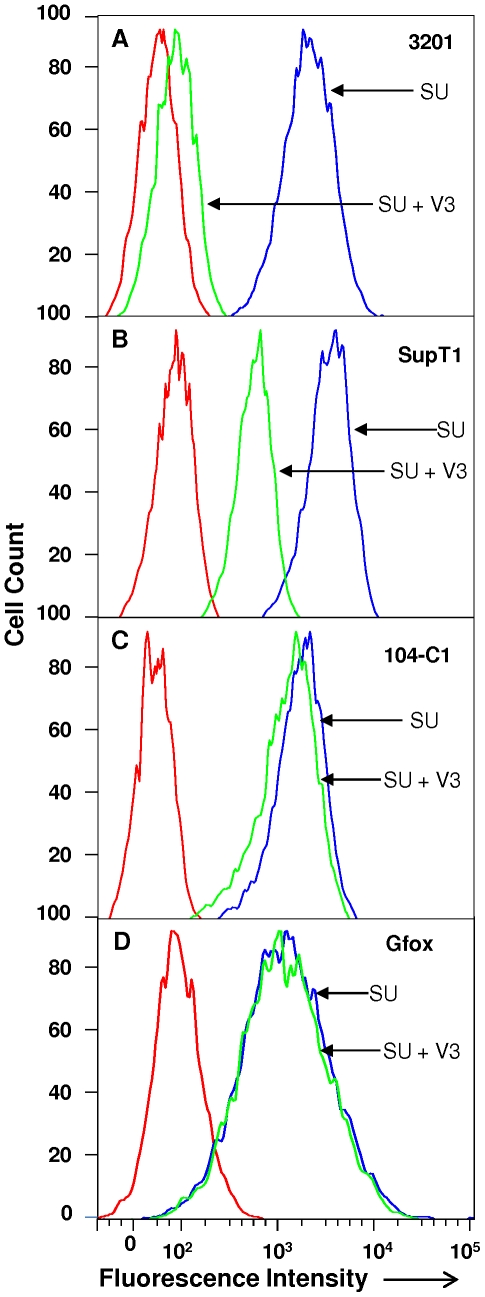
Inhibition of SU-Fc binding to 3201, SupT1, 104-C1 and Gfox cells by His-tagged V3 peptide. The peptide was pre-incubated with the cells for 1 h before the addition of SU-Fc to cells and incubated for another 1 h at room temperature. SU-Fc binding was detected by anti-Fc antibody and analyzed by FACS. V3 peptide blocks SU-Fc binding to 3201 and SupT1 cells, but has little effect on 104-C1 and Gfox cells. Results are representative of three independent determinations.

Our previous study using SU-Fc adhesins with deletion mutants of V2, V3, V4 or V5 showed that SU-Fc mutants with deletion of V2, V4 or V5 retained the ability to bind CXCR4. In contrast, the V3-deleted SU-Fc failed to bind 3201 cells [36]. Thus, the V3 region is critical for SU binding to CXCR4. Here, we utilized the His-tagged V3 peptide (Figure 3) to directly confirm the role of V3 region for CXCR4 binding in competition studies (Figure 4). The sequence of the V3 peptide and the linear binding epitopes for six anti-V3 antibodies are shown on Figure 3. FACS analysis showed that the V3 peptide could antagonize full-length SU-Fc binding to 3201 cells. At a comparable concentration as SU, the V3 peptide almost completely blocked the SU binding to 3201 cells (Figure 4A), and markedly reduced the binding of SU to human CXCR4 on SupT1 cells (Figure 4B). The peptide had negligible effect on SU binding to 104-C1 (Figure 4C) or Gfox cells (Figure 4D) (i.e., to CD134).

### Construction and expression of FIV-PPR SU-Fc mutants

**Figure 5:**
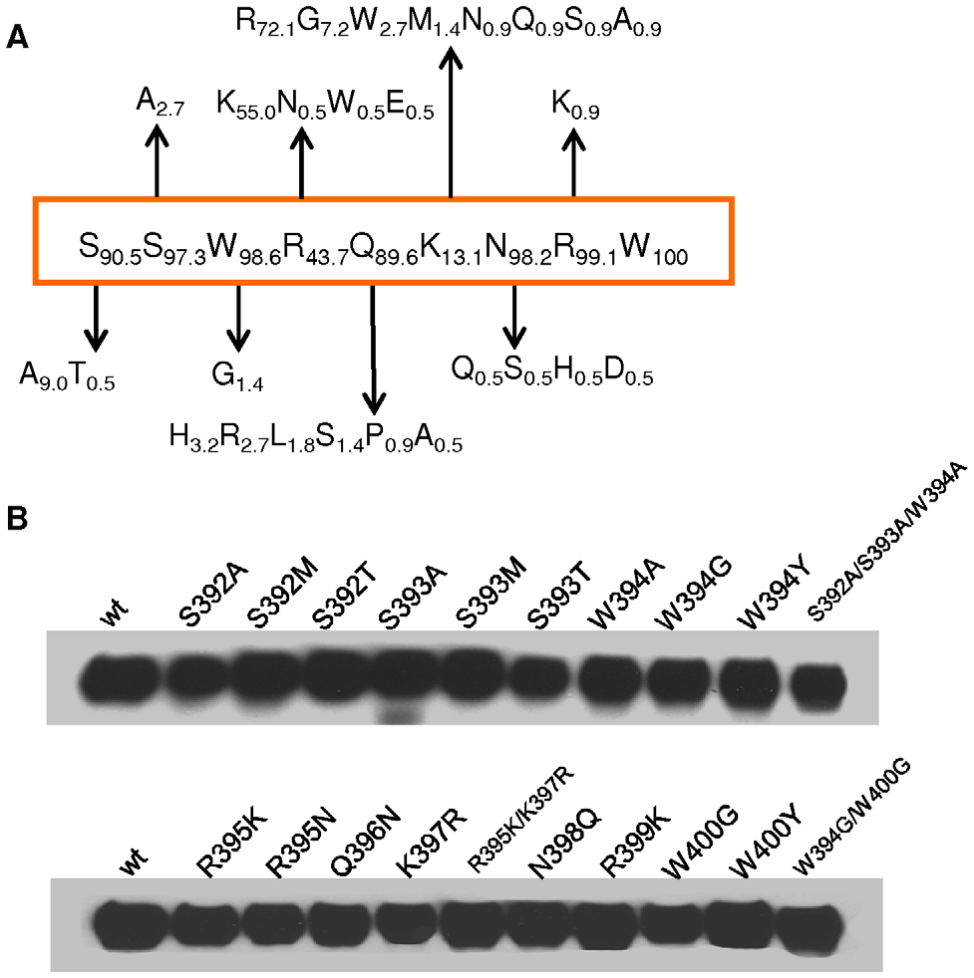
(**A**)**. Sequences and amino acid percent frequency of the “N44 region” of FIV isolates.** The number next to each residue indicates the frequency at which the corresponding residue is found among over 200 FIV envelope sequences published in Genbank. (**B**) **Expression of FIV SU-Fc with amino acid substitutions.** FIV SU-Fc mutants were generated by site-directed mutagenesis, expressed by stable transfection of CHO-K1 cells and batch purified from cell supernatants by affinity chromatography over protein A-Sepharose. 100 ng of SU-Fc (wt and mutants) were subjected to SDS-PAGE under reducing conditions and reacted with HRP-conjugated anti-human IgG1antibody. The labels are located above in which amino acid substitutions have been introduced, and the resulting mutants were assessed for abilities to bind CXCR4 and CD134.

Our analyses by using V3 deletion mutants, V3-specific mAbs, and V3 peptide strongly support the involvement of SU V3 in the FIV-CXCR4 interaction. We next sought to determine the minimal domain within the V3 region required for SU-CXCR4 interaction. Since a peptide as short as nine amino acids (SSWKQRNRW) designated N44, was potent in blocking SU binding [36], we evaluated a number of amino acid substituted mutants in the N44 region of FIV PPR SU to further reveal the key residues. Our mutagenic analysis employed a series of conservative or non-conservative changes, and so was used to assess the binding phenotypes induced by residues present in other FIV isolates (Figure 5A). In addition, either alanine substitutions or mutations likely to disrupt (non-conserved replacement) or imitate (conserved alteration) interactions were substituted (Figure 5B). SU-Fc constructs were generated by site-directed mutagenesis and soluble proteins were produced from CHO cell lines. All purified SU-Fc proteins were quantified by ELISA, and then subjected to SDS-PAGE and western blotting (using anti-human IgG1 Fc antibody) to confirm the expected size and relative quantitation of each mutant (Figure 5B).

### Fine mapping of the V3 region required for interaction with CXCR4

Binding affinity assays by FACS were performed using 3201, SupT1, and 104-C1 cells as described as “Material and Methods”. The value for binding of wild type SU-Fc to cells was arbitrarily set to 100%, and the binding data for specifically constructed mutants were normalized to this value. The results are summarized in Table 1, Table 2, Table 3.

**Table 1:** Effects of Amino Acid Substitutions in SU on CXCR4-binding Ability in 3201 Cells.

PPR SU variants	AMD 3100			
	-	0.03 µg/ml	0.1 µg/ml	0.3 µg/ml
Mock	0.1±0.1	-	-	-
Wild type	100.0±0.0	33.6±2.8	9.0±2.1	1.0±0.6
S392A	102.7±0.1	36.2±10.5	9.2±3.8	1.0±0.0
S392M	7.2±0.1	0.9±0.0	0.1±0.0	0.1±0.1
S392T	41.3±3.9	5.4±3.6	0.5±0.0	0.3±0.0
S393A	109.5±8.8	56.2±1.0	25.7±3.8	6.5±0.2
S393M	0.2±0.1	0.0±0.1	0.0±0.1	0.4±0.4
S393T	74.1±6.0	28.0±7.0	6.0±0.4	0.4±0.0
W394A	63.4±10.9	18.2±0.5	2.7±0.4	0.2±0.1
W394G	52.0±7.3	16.0±1.2	1.7±0.0	0.0±0.0
W394Y	63.4±1.6	16.8±3.4	4.2±0.0	0.2±0.0
S392A/S393A/W394A	35.2±9.3	4.1±0.6	0.2±0.0	1.0±2.0
R395K	103.7±2.0	32.3±6.4	11.6±4.5	3.5±0.3
R395N	6.5±0.7	1.3±0.3	0.4±0.2	0.2±0.1
Q396N	97.6±2.6	38.8±6.3	12.4±1.6	1.4±0.1
K397R	92.0±1.1	21.1±9.2	7.1±3.8	1.6±0.0
R395K/K397R	104.9±6.1	54.4±6.2	21.4±1.4	2.6±0.6
N398Q	0.4±0.0	0.7±0.7	0.2±0.1	0.2±0.0
R399K	0.0±0.1	0.0±0.2	0.0±0.2	0.1±0.1
W400G	5.4±1.1	0.2±0.1	0.0±0.1	0.0±0.1
W400Y	88.6±3.5	28.8±4.5	9.2±2.8	1.0±0.6
W394G/W400G	3.6±0.6	3.5±1.2	1.9±0.2	3.8±0.3

a. Binding of FIV PPR SU-Fc of wild type and mutants to 3201 was analyzed by FACS in the presence and absence of AMD3100. Cells were incubated with SU-Fc of wild type or mutants for 1 h at the room temperature or pre-treated with AMD3100 for 0.5 h before the addition of SU-Fc. Results are indicated as mean±SD of three independent determinations. Values for mutants are percentages of the mean fluorescence intensity of wild type SU, which is regarded as 100%.

b. Values in bold represent loss of specific antibody reactivity to a given mutant.

**Table 2:** Effects of Amino Acid Substitutions in SU on CXCR4-binding Ability in SupT1 Cells.

PPR SU variants	AMD 3100			
	-	0.01 µg/ml	0.03 µg/ml	0.1 µg/ml
Mock	0.1±0.0	-	-	-
Wild type	100.0±0.0	61.4±9.8	31.0±9.8	8.5±6.8
S392A	67.0±0.2	46.8±8.0	14.2±0.4	0.9±0.2
S392M	0.2±0.1	0.0±0.0	0.0±0.0	0.0±0.0
S392T	2.2±0.3	0.7±0.2	0.6±0.0	0.5±0.1
S393A	93.6±1.0	60.7±1.3	18.7±4.6	1.7±1.1
S393M	0.2±0.1	0.1±0.0	0.1±0.0	0.2±0.0
S393T	46.2±0.6	16.5±0.2	1.5±0.5	0.2±0.0
W394A	15.8±3.9	3.7±1.2	0.6±0.1	0.3±0.1
W394G	9.6±0.5	2.7±0.7	0.3±0.0	0.2±0.1
W394Y	32.5±2.1	14.1±0.2	1.2±0.4	0.4±0.0
S392A/S393A/W394A	6.0±2.6	2.5±1.9	1.0±0.6	0.5±0.4
R395K	99.2±1.2	67.3±1.7	38.3±0.7	6.7±2.2
R395N	7.8±0.4	2.1±0.9	0.2±0.2	0.1±0.0
Q396N	84.2±5.2	57.7±2.2	29.8±3.5	4.1±0.6
K397R	64.2±2.3	39.1±5.0	19.7±0.4	3.0±3.2
R395K/K397R	103.8±3.6	75.8±0.4	45.5±5.8	16.3±0.6
N398Q	0.2±0.0	0.4±0.4	0.1±0.0	0.2±0.0
R399K	0.2±0.3	0.2±0.3	0.0±0.0	0.0±0.0
W400G	0.2±0.0	0.3±0.0	0.2±0.0	0.2±0.0
W400Y	91.3±2.1	60.9±4.7	20.1±5.6	1.7±1.0
W394G/W400G	9.1±0.8	2.7±0.10	0.8±0.6	0.7±0.6

a. Binding of FIV PPR SU-Fc of wild type and mutants to SupT1 was analyzed by FACS in the presence and absence of AMD3100. Cells were incubated with SU-Fc of wild type or mutants for 1 h at the room temperature or pre-treated with AMD3100 for 0.5 h before the addition of SU-Fc. Results are indicated as mean±SD of three independent determinations. Values for mutants are percentages of the mean fluorescence intensity of wild type SU, which is regarded as 100%.

b. Values in bold represent loss of specific antibody reactivity to a given mutant.

**Table 3:** Effects of Amino Acid Substitutions in SU on CD134-binding Ability in 104-C1 Cells.

PPR SU variants	AMD 3100	
	-	+
Mock	0.4±0.0	-
Wild type	100.0±0.0	92.4±3.6
S392A	68.6±7.8	52.7±0.9
S392M	50.3±9.4	53.3±2.6
S392T	60.8±3.2	55.3±2.5
S393A	98.4±4.9	91.0±1.8
S393M	77.9±7.8	75.6±0.1
S393T	81.0±7.8	78.6±4.6
W394A	82.5±7.9	76.6±6.1
W394G	77.0±4.0	73.4±1.3
W394Y	81.1±7.5	75.9±9.8
S392A/S393A/W394A	65.1±9.1	64.2±10.7
R395K	100.4±8.1	81.1±9.5
R395N	65.7±3.7	67.6±6.0
Q396N	65.2±3.3	59.8±6.4
K397R	73.454±5.5	64.2±4.3
R395K/K397R	87.3±2.5	73.9±1.2
N398Q	69.0±6.7	64.8±7.9
R399K	84.6±0.0	84.0±3.8
W400G	56.5±0.2	61.9±7.4
W400Y	74.7±0.7	78.1±1.6
W394G/W400G	52.3±8.4	57.1±4.6

Binding of FIV PPR SU-Fc of wild type and mutants to 104-C1 was analyzed by FACS in the presence and absence of AMD3100. Cells were incubated with SU-Fc of wild type or mutants for 1 h at the room temperature or pre-treated with AMD3100 for 0.5 h before the addition of SU-Fc. Results are indicated as mean±SD of three independent determinations. Values for mutants are percentages of the mean fluorescence intensity of wild type SU, which is regarded as 100%.

As seen from table1 (using 3201 cells) or table 2 (using SupT1 cells), the binding of FIV PPR SU-Fcs to 3201 and SupT1 cells is specific for CXCR4 since it can be inhibited by AMD3100 in a dose-dependent manner. In 3201 cells, alanine substitutions introduced into residues S392 and S393 did not significantly affect the binding of SU mutants to CXCR4, whereas introduction of methionine (S392M or S393M) dramatically reduced CXCR4 binding. By contrast, the more conservative introduction of threonine at the same sites affected CXCR4 binding much less. W394A partially abolished the CXCR4 binding affinity. A triple-alanine substituted mutant S392A/S393A/W394A decreased the binding efficiency to less than 40%. The mutant W394G or W394Y also reduced the CXCR4-binding affinity at a similar level to W394A. Although W400 and W394 are similarly conserved among FIV isolates, mutant W400G, together with W394G/W400G, exhibited specific defects in the ability to bind CXCR4, which indicates that W400 is more sensitive than W394 to the same substitution of glycine. However, when W400 was changed to Y400, the reduction of binding affinity was minimal. Overall, the results suggest that residue W400 requires some steric effect for CXCR4 binding which is accommodated by aromatic amino acids in general. Amino acid substitutions by the same kinds of basic residues kept the same binding capacities as wild type or binding was mildly enhanced, as seen with R395K, K397R, and R395K/K397R. An exception was an R399K substitution, which completely abolished SU binding. However, when basic residue R395 was replaced by a non-basic residue, R395N, the mutant lost the binding efficiency. Interestingly, mutant SU with substitution of Q396N retained the binding affinity to CXCR4, whereas, alteration of N398Q completely abrogated CXCR4 binding ability, although both residues were replaced by the same character of amino acid residue. Based on the data, we can conclude that R395, K397, N398, and R399 are important for CXCR4 binding. S392, S393, W394 and W400 also affect the binding, but the effects at these latter positions depend on the nature of the amino acid substitution. None of the amino acid substitutions significantly increased SU binding to CXCR4, although the mutant of S393A caused a slight enhancement of approximately 10%.

Similar trends were noted in the binding efficiencies of the panel of mutant SU to human CXCR4 on SupT1 cells (Table 2). However, certain sites caused greater reductions in binding to the human homologue than to feline CXCR4 (compare values, Tables 1 and 2). In particular, S392T and W394G mutations caused greater loss of binding to human than feline CXCR4 (bold values, Table 2), with reductions of 90–100% compared to 40–60% reduction to feline CXCR4 (Table 1).

Amino acid residues critical for CXCR4 binding were less crucial for interactions with the primary binding receptor CD134, i.e., the above amino acid substitutions had a more global effect on binding to CD134 on 104-C1 cells, with approximately 20-50% reduction in SU binding irrespective of amino acid substitutions at sites critical for CXCR4 binding (Table 3). Therefore, it appears that the V3 loop, particularly its “N44 region”, is directly required for interaction of SU with the entry receptor CXCR4 but not with the binding receptor CD134. The results indicate that the binding of CD134 is heavily dependent on the overall conformation of SU rather than on a short contiguous epitopes as with CXCR4.

### Infectivity assay using pseudotyped FIV

**Figure 6:**
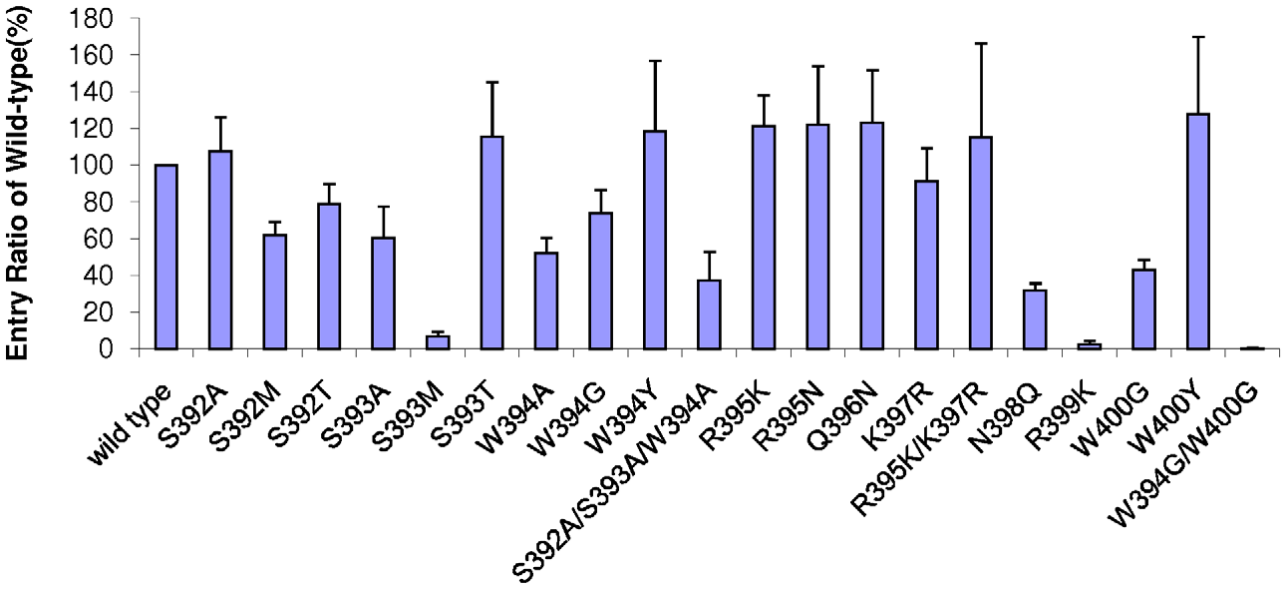
Virus entry analysis of β-gal-expressing pseudovirions with SU of wild type or mutants in Gfox cells. Supernatants from 293T cells transfected with constructs expressing either wild type or mutant SUs were analyzed for RT activity 48 h after transfection and RT values were normalized for wt and mutants. Pseudovirions were then used in single-round infections of Gfox cells. pCFIV vector pseudotyped with a deleted envelope (ΔEnv) was used to assess the degree of viral entry. A β-gal assay was performed 48 h after infection. Values for mutants are percentages of the mean relative luminescence units of wild type SU set at 100%. Results are means±SD for three independent determinations.

To confirm that SU binding is directly related to the entry efficiency, a virus entry assay was performed in Gfox cells. β-galactosidase-expressing vectors were pseudotyped with FIV-PPR SUs replaced by the same amino acid substitutions described above and used for production of virions in 293T cells. After normalizing the virus supernatants for RT activity, the pseudovirions were then used in single-round virus entry assays in Gfox cells [6]. Introduction of alanine at S392 had no effect, but the same substitution at S393 or W394 (or the triplet S392/S393/W394) partially abrogated the ability of the mutant SU to facilitate virus entry into Gfox cells (entry ratios between 35%–60%, compared with wild type, Figure 6). The alteration of S393M essentially abrogated virus entry while S392M retained modest entry capacities. S392T and S393T mutants entered the cells at levels comparable to wild type SU, with entry ratios of 80% and 115%, respectively. The alteration of W394G decreased the efficiency of viral entry in Gfox cells, whereas, W394Y infected at 118% of wild type levels. The mutants R395K, R395K/K397R, Q396N and K397R infected at wild type levels, with a slight increase. However, N398Q and R399K caused almost complete loss of ability to enter the cells. The substitution of W400G and W394G/W400G blocked the ability of the mutant SU to facilitate virus entry into Gfox cells, while mutant W400Y could enter the cells. Surprisingly, although the mutant of R395N exhibited a severe reduction in CXCR4-binding ability, the change in this residue exerted only little effect on virus entry. Another exception was S393A, which retained SU binding abilities (Table 1), but performed poorly in the entry assay (Figure 6). Compared with the CXCR4 binding affinities, S392M, S392T, W394Y and W400G increased entry capacities to some extent, but they still had weak entry efficiencies or had a mild change.

### Structural mapping of V3-specific monoclonal antibodies to SU

To further characterize the binding sites of V3-specific neutralizing monoclonal antibodies (mAbs) generated by immunizing mice with SU-Fc [36], [42], [43], ELISA assays were performed with the same set of SU-Fc proteins used in the binding studies (Table 4, Table 5). All of the mutants interacted with the SU1-5 and SU1-10 mAbs comparably to that of the wild-type SU, consistent with the binding epitopes of these antibodies being outside N44 region. SU1-7 was a conformation dependent antibody, which could not be recognized by mutants R399K, W400G and W394GW400G. The affinity of SU 1-30 for all of W394 mutants was dramatically reduced, while the other mutants bound similarly to wild type SU, indicating that W394 is a key residue for the epitope recognized by SU 1-30 (Table 4). W400 was important for the recognition of SU2-4 and SU2-5. Also, two mutants with the change of tryptophan to glycine (W400G and W394G/W400G) were not recognized by SU2-4 or SU2-5. However, the binding abilities of these antibodies were 45–60% restored when this residue was replaced by tyrosine, a similar situation that had been observed in the binding studies. The binding affinity of SU2-10 for the W400G and W394GW400G was modestly reduced, indicating W400 also had some effect on the recognition of the antibody (Table 5). These results suggest that the recognition of V3 antibodies for some mutants is specific and neutralizing antibody-sensitive epitopes are located in the CXCR4 binding sites. Highly conserved residues W394 and W400 play an important role in the recognition by antibodies. The findings emphasize that FIV neutralizing antibodies recognize the CXCR4 binding region of SU or epitopes or in very close proximity to this domain.

**Table 4:** Mapping of binding sites of V3-specific SU MAbs by ELISA, using wild type and mutated SU-Fc proteins.

PPR SU variants	V3 antibodies			
	SU1-5	SU1-7	SU1-10	SU1-30
Wild type	100.0±0.0	100.0±0.0	100.0±0.0	100.0±0.0
S392A	84.8±3.8	85.5±3.4	89.7±2.4	98.6±8.0
S392M	82.0±2.2	89.0±4.7	86.0±6.8	98.0±3.4
S392T	85.2±3.3	88.8±3.5	90.2±7.2	90.4±6.7
S393A	79.8±2.2	96.4±5.6	95.8±4.1	80.8±4.0
S393M	89.6±5.0	93.8±3.3	92.8±1.0	89.4±6.6
S393T	87.7±9.0	91.5±3.4	99.0±3.2	86.1±7.3
W394A	84.8±1.5	94.8±4.9	90.6±5.5	23.5±0.7
W394G	82.8±2.6	95.4±5.4	94.6±3.8	9.0±5.4
W394Y	84.4±6.0	94.6±0.2	93.1±3.3	34.8±4.9
S392A/S393A/W394A	74.7±2.2	88.0±6.0	84.0±7.1	13.2±1.0
R395K	89.6±4.9	95.6±3.8	88.6±3.0	98.0±5.5
R395N	88.6±5.7	94.5±8.7	84.9±2.0	90.5±4.6
Q396N	93.6±5.2	94.6±2.8	86.6±3.0	93.4±4.4
K397R	100.0±5.6	91.5±2.9	96.8±5.4	90.5±1.2
R395K/K397R	91.6±4.8	89.2±7.6	92.8±8.6	93.1±7.8
N398Q	96.1±3.0	96.8±0.9	93.2±1.2	61.3±2.4
R399K	99.6±4.8	9.9±2.2	99.8±6.6	91.1±6.6
W400G	79.1±2.8	4.8±0.0	87.9±2.7	75.2±1.5
W400Y	84.6±7.7	58.0±3.0	84.0±5.3	80.0±6.4
W394G/W400G	79.0±5.2	7.5±0.2	78.8±9.0	11.5±2.2

a. Binding of FIV SU-Fc of wild type and mutants to V3-specific MAbs detected by ELISA. V3-specific MAbs were incubated with SU-Fc of wild type or mutants for 1 h at the room temperature. For measurement of the binding of antibodies to the wild type and mutant SU-Fc, various dilutions of SU-Fc were performed in a pilot experiment to ensure that binding occurred in the linear range of the assay. Results are indicated as mean±SD of three independent determinations. Values for mutants are percentages of the mean optical density value of wild type SU, which is regarded as 100%.

b. Values in bold represent loss of specific antibody reactivity to a given mutant.

**Table 5:** Mapping of binding sites of V3-specific SU MAbs by ELISA, using wild type and mutated SU-Fc proteins.

PPR SU variants	V3 antibodies		
	SU2-4	SU2-5	SU2-10
Wild type	100.0±0.0	100.0±0.0	100.0±0.0
S392A	91.2±7.0	95.4±3.8	96.1±6.2
S392M	95.8±7.6	95.7±0.5	97.7±2.7
S392T	93.6±0.8	90.5±3.5	84.7±4.0
S393A	99.5±8.6	95.6±3.1	92.5±5.4
S393M	86.5±0.8	98.4±5.8	91.8±6.2
S393T	92.8±3.8	91.0±6.7	94.7±6.3
W394A	96.0±4.1	97.4±6.2	92.0±5.0
W394G	85.8±5.2	91.6±6.5	89.6±5.3
W394Y	90.4±6.7	96.1±4.2	88.5±4.2
S392A/S393A/W394A	81.1±5.5	86.5±3.0	84.5±6.9
R395K	90.6±6.1	86.0±3.2	84.3±4.9
R395N	72.3±5.2	76.9±3.7	85.4±2.7
R395K/K397R	94.8±7.6	94.2±1.4	100.8±2.1
Q396N	95.5±7.3	95.9±6.0	97.9±2.8
K397R	84.2±3.6	95.1±1.9	89.5±6.9
N398Q	92.6±9.2	98.9±4.2	92.2±4.3
R399K	87.5±6.5	95.2±8.1	100.9±9.0
W400G	5.4±0.7	7.9±2.0	52.8±2.6
W400Y	66.4±1.3	43.5±3.1	63.7±5.6
W394G/W400G	5.7±0.9	13.5±3.1	42.8±3.7

a. Binding of FIV SU-Fc of wild type and mutants to V3-specific MAbs detected by ELISA. V3-specific MAbs were incubated with SU-Fc of wild type or mutants for 1 h at the room temperature. For measurement of the binding of antibodies to the wild type and mutant SU-Fc, various dilutions of SU-Fc were performed in a pilot experiment to ensure that binding occurred in the linear range of the assay. Results are indicated as mean±SD of three independent determinations. Values for mutants are percentages of the mean optical density value of wild type SU, which is regarded as 100%.

b. Values in bold represent loss of specific antibody reactivity to a given mutant.

## Discussion

Our previous studies defined a nine-amino acid domain at the tip of the V3 loop of FIV SU as critical for the interaction with CXCR4 [36]. In this report, we further define the key amino acid residues required for CXCR4 binding and establish the relationship between binding and entry. Site-directed mutagenesis was employed to introduce amino acid substitutions in the N44 region with residues that are found in other FIV isolates as well as changes that may influence CXCR4 binding. The panel of mutants was further utilized to define residues critical for neutralizing antibodies that recognize the V3 domain. In addition, using the binding assay in conjunction with a panel of receptor-expressing cell lines allowed simultaneous study of SU binding to CXCR4 and CD134. 3201 cells lack CD134 and heparan sulfate proteoglycans (HSPG) expression, so the use of 3201 in binding assays allows the evaluation of specific SU-CXCR4 interactions [41]. The 104-C1 continuous cell line is CD134^high^ and CXCR4^low^, which provides a venue to assess SU-CD134 interactions. Human CXCR4-expressing SupT1 cells were used to confirm the interactions of SU and CXCR4, based on the agreement of the high degree of homology between CXCR4 of feline and human origins [8]. Interestingly, human CXCR4 is a more efficient receptor for FIV than either feline CXCR4 or rat CXCR4 and was in fact employed in the original study that showed CXCR4 was the entry receptor for FIV [11].

Our results demonstrate several amino acid residues in the V3 loop make key contributions to CXCR4 binding. These key positions primarily consist of basic and aromatic residues. For HIV-1, some basic residues on the V3 loop likely contribute to electrostatic interactions and the formation of specific salt bridges with CXCR4 [35], [44], which is acidic at the N-terminus and in extracellular loops [13]. In addition, conserved hydrophobic residues in the V3 loop of HIV gp120, especially at the tip/crown of HIV V3, have been shown to contribute to CXCR4 binding [35]. Similarly, the N44 region in the FIV V3 loop includes three basic residues and two aromatic tryptophan residues among over 200 FIV envelope sequences published in Genbank. Essentially invariant in the majority of FIV envelope sequences, these three basic residues R395, K397, and R399 exhibit a limited number of conservative substitutions. Arginine or lysine is found at residues 395 and 397, although some non-charged or negative-charged residues are also found at the analogous position (Figure 5A). Consistent with a role for basic residues in CXCR4 binding, the mutants R395K, K397R, and R395K/K397R kept the same binding capacities as wild type or enhanced lightly in 3201 cells. In contrast, asparagine substitution introduced into R395 significantly blocked the binding of SU to CXCR4, indicating that electrostatic attraction plays an important role for CXCR4 binding with this residue. To our surprise, mutant R395N did not prevent virus entry into the Gfox cells, in spite of the observation that this mutant markedly reduced CXCR4 binding. We assume that there is some difference between dimeric SU-Fc in the context of a dimeric immunoadhesin and trimeric SU on the virus particle. Thus, the alteration of R395N in the V3 loop of FIV PPR allows this virus to utilize CXCR4 for entry into target cells under certain circumstances. Interestingly, arginine at residue 399 is highly conserved and is more important for CXCR4 binding, with even a conservative substitution of R399K resulting in disruption of CXCR4 binding and abrogation of virus entry. One interpretation of the results is that the loss of favorable CXCR4 interactions with this residue has been impacted by a steric effect much more than the electrostatic attraction.

Among the three basic residues are two polar uncharged amino acid residues, Q396 and N398. Of interest, substitution of Q396N maintained the binding affinity to CXCR4, whereas, replacement of N398Q dramatically abolished CXCR4 binding. Thus, it appears that steric effects have a substantial influence at these two residues with intolerance to conserved amino acid changes.

Two highly conserved tryptophan residues at positions 394 and 400 are found with the frequency of 98% (glycine is 2%) and 100%, respectively. When glycine was introduced into these two positions separately or combined, the mutant SU-Fc of W394G partially reduced the binding ability, whereas, W400G and W394G/W400G mutants were significantly attenuated. Moreover, the latter two mutants were not recognized efficiently by the SU1-7, SU2-4 and SU2-5 mAbs, consistent with previous studies indicating the involvement of W400 in the epitopes for the antibodies [36], [42], [43]. However, some tyrosine substitutions, although involving these two highly conserved positions, did not significantly modify the binding affinity to CXCR4 or capacity to enter Gfox cells. It appears that aromatic residues have a similar steric effect so that the conformation of V3 loop is preserved by a conservative substitution.

Other positions were relatively tolerant to conserved amino acid changes, but not to changes that substantially alter amino acid character. Alanine substitution for serine at positions 392 and 393 did not abolish SU binding to CXCR4, but the change to methionine dramatically reduced CXCR4 binding in both 3201 and SupT1 cells. When triple-alanine substitutions were introduced into positions 392, 393 and 394, the binding efficiency decreased to less than 40%, which suggests that these residues may contribute directly or indirectly to maintenance of a continuous surface for CXCR4 binding. Correspondingly, the effect of the threonine substitution at position 392 on CXCR4 binding was much more severe than that of alteration at position 393. Thus, serine at 392 may be more important for CXCR4 binding than at 393.

The present studies also show that the same set of N44 region mutants did not abrogate binding to the primary binding receptor CD134 on 104-C1 cells. Rather, all changes had a negative impact on CD134 binding. The results indicate that binding of SU to CD134 is highly conformation dependent, consistent with the inability in this study or in previous efforts [36] to define one distinct binding domain. Since the Gfox cell line expresses both CD134 and CXCR4, it may well be that some of the reduction observed in the CD134 binding assay for some mutants may also contribute to reduced infectivity observed in figure 6. However, the reduction of infectivity correlates well with loss of CXCR4 binding; i.e., we do not see loss in infectivity with any mutant that did not lose CXCR4 binding ability, which one would expect if the mutation primarily impacted on CD134 association and therefore caused loss in infectivity.

Although the V3 loop of SU contains the main determinants for FIV SU binding to CXCR4, we do not exclude the possibility that the V2 and V4 loops in some way contribute to the interaction with CXCR4. X-ray crystallography or nuclear magnetic resonance analysis needs to be carried out in order to further define structural involvement in receptor interactions. It will also be of interest to determine whether the N44 region of SU interacts with the extracellular loop-2 (ECL-2) or other parts of CXCR4.

The present study demonstrates the general features of the CXCR4-binding surface of SU and the relatively subtle adjustments in the V3 loop of FIV isolates that can impact on receptor binding. The identification of structures important for receptor binding of FIV isolates should help in defining targets for blocking virus entry. Moreover, the information contributes valuable knowledge for the use of the FIV/cat system as a small animal model for HIV/AIDS.

## Materials and Methods

### Cell lines, virus and reagents

The interleukin-2 (IL-2)-independent feline lymphoma cell line 3201 was obtained from William Hardy (Sloan-Kettering Memorial Hospital, NY). The primary IL-2-dependent T-cell line 104-C1 is a clone isolated from feline PBMCs by limiting dilution and was a gift from Chris K. Grant (Custom Monoclonal Antibodies, Intl.). CrFK cells were obtained from the ATCC (Rockville, Md.). Gfox cells are CXCR4^+^ Crandell feline kidney (CrFK) cells stably transfected with feline CD134 and thus productively infectable by field strains of FIV [6], [45]. CHO-K1 and 293T cells were obtained from the American Type Culture Collection (Rockville, MD). Propagation of the above cell lines was performed as previously described [6], [9], [41], [46], [47]. Human T-lymphocytic SupT1 cells expressing CXCR4 were acquired from Bruce Torbett (The Scripps Research Institute) and cultivated in DMEM medium supplemented with 10% fetal bovine serum (FBS, Invitrogen, Carlsbad, CA), 2 mM L-glutamine (Sigma, St. Louis, MO), non-essential amino acids (Sigma), Penicillin- Streptomycin 100 u/ml and 100 µg/ml respectively (Invitrogen). The FIV strain used in the present study was FIV-PPR, a molecularly cloned clade A primary isolate of the FIV San Diego isolate [28]. Human specific anti-CXCR4 antibody 12G5 [38]–[40] was obtained from BD Biosciences (Franklin Lakes, NJ), feline anti-CXCR4 monoclonal antibody was purchased from R&D Systems, Inc (Minneapolis, MN), which is highly specific for feline CXCR4. CrFK cells transfected with feline CXCR4 could be stained brightly with the feline anti-CXCR4 antibody, while CrFK cells transfected with human CXCR4, were not stained by this antibody, supporting a high degree of antibody specificity. In addition, the staining for feline CXCR4 transfected CrFK cells could be inhibited by feline CXCR4 siRNA treatment (see Figure S1). Rabbit anti-feline CD134 antibody was homemade [6]. Rabbit anti-His antibody was obtained from Santa Cruz Biotechnology, Inc (Santa Cruz, CA). V3-specific monoclonal antibodies (mAbs) were generated by immunizing mice with SU-Fc, as reported previously [36], [42], [43]. Rigorous tests for relative affinity have not been carried out, but an evaluation of the antibody neutralizing activity was performed, with IC_50_ values around 10 µg/ml. AMD3100 was purchased from Sigma.

### Construct, expression, and purification of His-tagged V3 peptide

The 41 aa sequence corresponding to V3 loop of FIV PPR was cloned by using primers 5′-GTGGATGGTGGAATCATATGGCCTATTATAACAG-3′ to insert an NdeI site on the 5′ side of V3, and 5′-CTCCTGGAATTCTCATTGCAAAAGTTTAATTATG-3′ to insert an EcoRI site on the 3′ side of V3. The completed PCR reaction was digested with DpnI for 1 hour at 37°C before gel purification by utilization of the Quick Gel Extraction Kit (Invitrogen). The V3 region was ligated into the TOPO cloning vector pCR2.1 (Invitrogen) and transformed into Top10 cells. Colonies were screened by using the same primers described as above, and colonies containing the expected sequence were midiprepped (Invitrogen). Then, pCR2.1 TOPO-V3 along with pET28a was cut with NdeI and EcoRI following New England Biolab's recommendations. The V3 insert and pET28a vector were gel purified post restriction digestion and ligated together before being transformed into Top10 for selection. V3 in frame with a 6x His-tag was selected and transformed into competent *Escherichia coli* Rosetta (DE3) pLysS cells (Novagen). Isopropyβ-D-thiogalactoside induced protein expression was scaled up, and purification was carried out by using three rounds of Ni-affinity chromatography.

### Recombinant SU proteins

Mutations were introduced into a pRSC-GS-SU-Fc expressor plasmid and performed by using the QuikChange site-directed mutagenesis strategy (Stratagene, La Jolla, CA) as recommended by the manufacturer. The presence of the desired mutations and the absence of any other mutation was confirmed by DNA sequencing. Mutated plasmids were then used for production of stable CHO-K1 cell lines, as previously described [48]. Single colonies with high expression of the desired Fc-tagged proteins were selected by single colony disk (Labcor Products,Inc, San Diego, CA). SU-Fc fusion proteins (adhesins) with the desired mutations or wild type SU-Fc from stable CHO cell supernatants were purified by affinity chromatography over protein A-Sepharose 4FastFLow beads (GE Healthcare, Piscataway, NJ) and eluted in a 100 mM citric acid and 150 mM NaCl solution (pH 2.1) into 100 µl 1 M Tris (pH 11.0). The elutions were then washed and concentrated three times with 1X PBS in a 100,000 MWC Amicon Ultra centrifugal filter device (Millipore, Bilerica, MA). After that, purified Fc-tagged fusion proteins were quantified using a human IgG ELISA quantitation kit (Bethyl Laboratories, Inc, Montgomery, TX), and the process was performed according to the manufacture and the data caculated by Sigmaplot. Finally, relative quantitation of proteins was confirmed by western blot analysis.

### Western blot analysis

100 ng of purified Fc-tagged fusion proteins was loaded on an 8 to 16% sodium dodecyl sulfate (SDS)-polyacrylamide gel (PAGE, Invitrogen) and transferred to a nitrocellulose membrane. The membrane was blocked with blotto for 30 minutes before the exposure to horseradish peroxidase (HRP) conjugated goat anti-human IgG (Fc- specific) antibody (MP Biomedicals, Solon, Ohio, 1∶1000 dilution) for 1 h at room temperature. Then, the membrane was washed with PBST (phosphate-buffered saline containing 0.05% Tween 20) for 10 minutes, 3 times, before a final rinse with water. The signal was detected by enhanced chemiluminescence using SuperSignal West Dura Extended Duration Substrate (Thermo Scientific, Rockford, IL).

### Flow cytometry analysis

Binding of SU-Fc fusion proteins or Fc (negative control) to the surfaces of 3201, 104-C1, or Sup-T1 cells was analyzed by flow cytometry, using a phycoerythrin-conjugated goat anti-human IgG1 Fc antibody (MP Biomedicals, Aurora, OH) and FLOWJO software (Tree Star, San Carlos, CA). CXCR4-specific binding was confirmed by pre-treatment of cells with the CXCR4 inhibitor, AMD3100 at the indicated concentration for 0.5 h at 25°C, followed by the incubation with SU-Fc fusion proteins for 1 h at 25°C. For His-tagged V3 peptide binding studies, the peptide was incubated at 25°C for 1 h, then competed with wild type SU-Fc fusion proteins for 1 h at 25°C. Receptor expression was measured by using anti-CXCR4 or CD134 antibody incubated with cells for 1 h at 25°C, followed by incubation with phycoerythrin-conjugated goat anti-mouse (CXCR4) or goat anti-rabbit (CD134) antibody for 45 min at 25°C.

### Virus entry assay

Cytomegalovirus_FIV hybrid vectors (pCFIV) [6], [45], [49] pseudotyped with either wild type FIV-PPR envelope (PPR Env) genes or mutants substituted by specific amino acids were cotransfected with a beta-galactosidase (β-gal)-expressing vector [6], [45], [49] in 293T cells. Two days later, viral supernatants were collected and each pseudovirion was assessed for the level of reverse transcriptase (RT). RT values were normalized before performing a single round infection assay in Gfox cells [45]. After 48 h of infection, β-gal activity was measured with the Tropix Galacto-Star chemiluminescent reporter gene assay (Applied Biosystems Inc, Bedford, MA) according to the manufacturer's guidelines.

### Micro-RT activity assay

Micro-RT activity assay was performed as previously described [9], [36], [48]. Briefly, 50 µl of cell-free supernatant together with 10 µl of lysis buffer (0.75 M KCl, 20 mM dithiothreitol, 0.5% Triton X-100) was incubated at room temperature for 10 minutes. Then, 40 µl of a mixture containing 125 mM Tris-HCl (pH 8.1), 12.5 mM MgCl_2_, 1.25 µg poly(rA)-poly(dT)_12–18_ (Amersham Biosciences, Piscataway, NJ) and 1.25 µCi of [^3^H]dTTP (DuPont, Boston, MA) was added to the sample followed by 2 h of incubation at 37°C. The measurement of RT activity was previously described [48].

### Enzyme-linked immunosorbent assay (ELISA)

Immulon II HB plates (Thermo, Milford, MA) were coated overnight with 1 µg of anti-V3 antibodies in PBS (pH 7.2). Then the plate washed twice with PBS and dried. 100 ng of purified SU-Fc fusion proteins of wild type or mutants was diluted in 100 µl of ELISA buffer (0.15 M NaCl, 0.05 M Tris-HCl, 1 mM EDTA, 3% bovine serum albumin fraction V, 3.5% fetal calf serum, and 0.05% Tween 20, pH 7.4), then added to appropriate wells. After 1.5 h incubation, the plate was washed three times with PBS and dried. HRP-conjugated goat anti-human IgG antibody diluted in ELISA buffer was added to every well and incubated for 1 h. After the same washing procedure, enzyme substrate reaction was performed for 10 min by using OPD as substrate, followed by the addition of stop solution of 2M H_2_SO_4_. The OD value was read at 493 nm using a microtiter plate reader. All operations were carried out at room temperature.

## Supporting Information

Figure S1 Specificity of anti-feline CXCR4 antibody. (A). CXCR4 expression on the cell surface was detected by FACS analysis in CrFK (left panel), feline CXCR4-transfected CrFK (middle panel), and human CXCR4-transfected CrFK (right panel) cells, respectively. (B). FACS analysis of the CXCR4 expression in the feline CXCR4-transfected CrFK cells with (left panel) or without (right panel) pretreatment with CXCR4-specific siRNA. Cells were stained with anti-feline CXCR4 antibody. Black line indicates background staining, blue or red line indicates CXCR4 staining.
(0.16 MB TIF)

## References

[1] Pedersen NC, Ho EW, Brown ML, Yamamoto JK (1987). Isolation of a T-lymphotropic virus from domestic cats with an immunodeficiency-like syndrome.. Science.

[2] Elder JH, Sundstrom M, de Rozieres S, de Parseval A, Grant CK (2008). Molecular mechanisms of FIV infection.. Vet Immunol Immunopathol.

[3] Podell M, Buck WR, Hayes KA, Gavrilin MA, Mathes LE (2002). Animal models of retroviral encephalopathies: feline model.. Curr Protoc Neurosci.

[4] Uhl EW, Martin M, Coleman JK, Yamamoto JK (2008). Advances in FIV vaccine technology.. Vet Immunol Immunopathol.

[5] Willett BJ, Hosie MJ (2008). Chemokine receptors and co-stimulatory molecules:unravelling feline immunodeficiency virus infection.. Vet Immunol Immunopathol.

[6] de Parseval A, Chatterji U, Sun P, Elder JH (2004). Feline immunodeficiency virus targets activated CD4+ T cells by using CD134 as a binding receptor.. Proc Natl Acad Sci U S A.

[7] Shimojima M, Miyazawa T, Ikeda Y, McMonagle EL, Haining H (2004). Use of CD134 as a primary receptor by the feline immunodeficiency virus.. Science.

[8] Willett BJ, Picard L, Hosie MJ, Turner JD, Adema K (1997). Shared usage of the chemokine receptor CXCR4 by the feline and human immunodeficiency viruses.. J Virol.

[9] de Parseval A, Elder JH (2001). Binding of recombinant feline immunodeficiency virus surface glycoprotein to feline cells: role of CXCR4, cell-surface heparans, and an unidentified non-CXCR4 receptor.. J Virol.

[10] Richardson J, Pancino G, Merat R, Leste-Lasserre T, Moraillon A (1999). Shared usage of the chemokine receptor CXCR4 by primary and laboratory-adapted strains of feline immunodeficiency virus.. J Virol.

[11] Willett BJ, Adema K, Heveker N, Brelot A, Picard L (1998). The second extracellular loop of CXCR4 determines its function as a receptor for feline immunodeficiency virus.. J Virol.

[12] Brelot A, Heveker N, Adema K, Hosie MJ, Willett B (1999). Effect of mutations in the second extracellular loop of CXCR4 on its utilization by human and feline immunodeficiency viruses.. J Virol.

[13] Picard L, Wilkinson DA, McKnight A, Gray PW, Hoxie JA (1997). Role of the amino-terminal extracellular domain of CXCR-4 in human immunodeficiency virus type 1 entry.. Virology.

[14] Rosenkilde MM, Gerlach LO, Jakobsen JS, Skerlj RT, Bridger GJ (2004). Molecular mechanism of AMD3100 antagonism in the CXCR4 receptor: transfer of binding site to the CXCR3 receptor.. J Biol Chem.

[15] Hatse S, Princen K, Vermeire K, Gerlach LO, Rosenkilde MM (2003). Mutations at the CXCR4 interaction sites for AMD3100 influence anti-CXCR4 antibody binding and HIV-1 entry.. FEBS Lett.

[16] Chabot DJ, Zhang PF, Quinnan GV, Broder CC (1999). Mutagenesis of CXCR4 identifies important domains for human immunodeficiency virus type 1 X4 isolate envelope-mediated membrane fusion and virus entry and reveals cryptic coreceptor activity for R5 isolates.. J Virol.

[17] Ling H, Usami O, Xiao P, Gu HX, Hattori T (2004). The N-terminal of the V3 loop in HIV type 1 gp120 is responsible for its conformation-dependent interaction with cell surface molecules.. AIDS Res Hum Retroviruses.

[18] Hartley O, Klasse PJ, Sattentau QJ, Moore JP (2005). V3: HIV's switch-hitter.. AIDS Res Hum Retroviruses.

[19] Rusche JR, Javaherian K, McDanal C, Petro J, Lynn DL (1988). Antibodies that inhibit fusion of human immunodeficiency virus-infected cells bind a 24-amino acid sequence of the viral envelope, gp120.. Proc Natl Acad Sci U S A.

[20] Wu L, Gerard NP, Wyatt R, Choe H, Parolin C (1996). CD4-induced interaction of primary HIV-1 gp120 glycoproteins with the chemokine receptor CCR-5.. Nature.

[21] Trkola A, Dragic T, Arthos J, Binley JM, Olson WC (1996). CD4-dependent, antibody-sensitive interactions between HIV-1 and its co-receptor CCR-5.. Nature.

[22] Dittmar MT, McKnight A, Simmons G, Clapham PR, Weiss RA (1997). HIV-1 tropism and co-receptor use.. Nature.

[23] Fouchier RA, Groenink M, Kootstra NA, Tersmette M, Huisman HG (1992). Phenotype-associated sequence variation in the third variable domain of the human immunodeficiency virus type 1 gp120 molecule.. J Virol.

[24] Cardozo T, Kimura T, Philpott S, Weiser B, Burger H (2007). Structural basis for coreceptor selectivity by the HIV type 1 V3 loop.. AIDS Res Hum Retroviruses.

[25] Morikawa S, Lutz H, Aubert A, Bishop DH (1991). Identification of conserved and variable regions in the envelope glycoprotein sequences of two feline immunodeficiency viruses isolated in Zurich, Switzerland.. Virus Res.

[26] Pancino G, Ellerbrok H, Sitbon M, Sonigo P (1994). Conserved framework of envelope glycoproteins among lentiviruses.. Curr Top Microbiol Immunol.

[27] Pancino G, Fossati I, Chappey C, Castelot S, Hurtrel B (1993). Structure and variations of feline immunodeficiency virus envelope glycoproteins.. Virology.

[28] Phillips TR, Talbott RL, Lamont C, Muir S, Lovelace K (1990). Comparison of two host cell range variants of feline immunodeficiency virus.. J Virol.

[29] Siebelink KH, Chu IH, Rimmelzwaan GF, Weijer K, Osterhaus AD (1992). Isolation and partial characterization of infectious molecular clones of feline immunodeficiency virus obtained directly from bone marrow DNA of a naturally infected cat.. J Virol.

[30] Sundaravaradan V, Das SR, Ramakrishnan R, Sehgal S, Gopalan S (2007). Role of HIV-1 subtype C envelope V3 to V5 regions in viral entry, coreceptor utilization and replication efficiency in primary T-lymphocytes and monocyte-derived macrophages.. Virol J.

[31] Sander O, Sing T, Sommer I, Low AJ, Cheung PK (2007). Structural descriptors of gp120 V3 loop for the prediction of HIV-1 coreceptor usage.. PLoS Comput Biol.

[32] Hwang SS, Boyle TJ, Lyerly HK, Cullen BR (1991). Identification of the envelope V3 loop as the primary determinant of cell tropism in HIV-1.. Science.

[33] Huang CC, Tang M, Zhang MY, Majeed S, Montabana E (2005). Structure of a V3-containing HIV-1 gp120 core.. Science.

[34] LaRosa GJ, Davide JP, Weinhold K, Waterbury JA, Profy AT (1990). Conserved sequence and structural elements in the HIV-1 principal neutralizing determinant.. Science.

[35] Basmaciogullari S, Babcock GJ, Van Ryk D, Wojtowicz W, Sodroski J (2002). Identification of conserved and variable structures in the human immunodeficiency virus gp120 glycoprotein of importance for CXCR4 binding.. J Virol.

[36] Sundstrom M, White RL, de Parseval A, Sastry KJ, Morris G (2008). Mapping of the CXCR4 binding site within variable region 3 of the feline immunodeficiency virus surface glycoprotein.. J Virol.

[37] McKnight A, Wilkinson D, Simmons G, Talbot S, Picard L (1997). Inhibition of human immunodeficiency virus fusion by a monoclonal antibody to a coreceptor (CXCR4) is both cell type and virus strain dependent.. J Virol.

[38] Willett BJ, Cannon CA, Hosie MJ (2002). Upregulation of surface feline CXCR4 expression following ectopic expression of CCR5: implications for studies of the cell tropism of feline immunodeficiency virus.. J Virol.

[39] Willett BJ, Hosie MJ, Neil JC, Turner JD, Hoxie JA (1997). Common mechanism of infection by lentiviruses.. Nature.

[40] Baribaud F, Edwards TG, Sharron M, Brelot A, Heveker N (2001). Antigenically distinct conformations of CXCR4.. J Virol.

[41] de Parseval A, Ngo S, Sun P, Elder JH (2004). Factors that increase the effective concentration of CXCR4 dictate feline immunodeficiency virus tropism and kinetics of replication.. J Virol.

[42] de Parseval A, Grant CK, Sastry KJ, Elder JH (2006). Sequential CD134-CXCR4 interactions in feline immunodeficiency virus (FIV): soluble CD134 activates FIV Env for CXCR4-dependent entry and reveals a cryptic neutralization epitope.. J Virol.

[43] Elder JH, Lin YC, Fink E, Grant CK (2010). Feline immunodeficiency virus (FIV) as a model for study of lentivirus infections: parallels with HIV.. Curr HIV Res.

[44] Moore JP, Sattentau QJ, Wyatt R, Sodroski J (1994). Probing the structure of the human immunodeficiency virus surface glycoprotein gp120 with a panel of monoclonal antibodies.. J Virol.

[45] de Parseval A, Chatterji U, Morris G, Sun P, Olson AJ (2005). Structural mapping of CD134 residues critical for interaction with feline immunodeficiency virus.. Nat Struct Mol Biol.

[46] Lerner DL, Grant CK, de Parseval A, Elder JH (1998). FIV infection of IL-2-dependent and -independent feline lymphocyte lines: host cells range distinctions and specific cytokine upregulation.. Vet Immunol Immunopathol.

[47] Sundstrom M, Chatterji U, Schaffer L, de Rozières S, Elder JH (2008). Feline immunodeficiency virus OrfA alters gene expression of splicing factors and proteasome-ubiquitination proteins.. Virology.

[48] de Parseval A, Lerner DL, Borrow P, Willett BJ, Elder JH (1997). Blocking of feline immunodeficiency virus infection by a monoclonal antibody to CD9 is via inhibition of virus release rather than interference with receptor binding.. J Virol.

[49] Johnston JC, Gasmi M, Lim LE, Elder JH, Yee JK (1999). Minimum requirements for efficient transduction of dividing and nondividing cells by feline immunodeficiency virus vectors.. J Virol.

