# Supplementary figures and images for: Fine Definition of the CXCR4-Binding Region on the V3 Loop of Feline Immunodeficiency Virus Surface Glycoprotein
Source: PLoS One. 2010 May 18;5(5):e10689. doi: 10.1371/journal.pone.0010689 (PMC2872658; doi:10.1371/journal.pone.0010689)

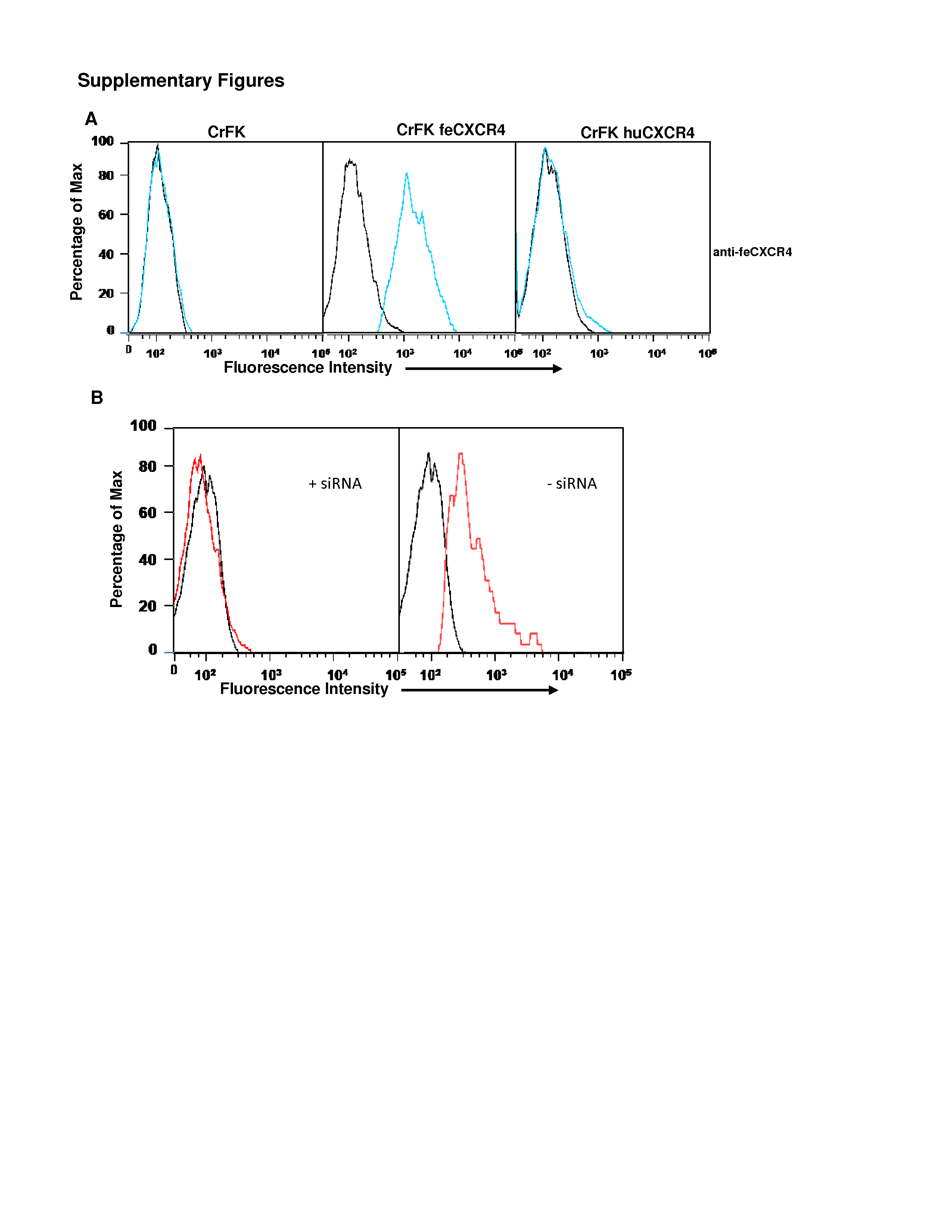

Supplement: Figure S1 — Specificity of anti-feline CXCR4 antibody. (A). CXCR4 expression on the cell surface was detected by FACS analysis in CrFK (left panel), feline CXCR4-transfected CrFK (middle panel), and human CXCR4-transfected CrFK (right panel) cells, respectively. (B). FACS analysis of the CXCR4 expression in the feline CXCR4-transfected CrFK cells with (left panel) or without (right panel) pretreatment with CXCR4-specific siRNA. Cells were stained with anti-feline CXCR4 antibody. Black line indicates background staining, blue or red line indicates CXCR4 staining. (0.16 MB TIF) [file pone.0010689.s001.tif]
